# Radiographic study of direct anterior approach hip arthroplasty: a 10–15 year follow-up of Chinese patients

**DOI:** 10.1186/s42836-024-00249-z

**Published:** 2024-05-03

**Authors:** Weilin Sang, Peng Lai, Xun Xu, Yu Liu, Jinzhong Ma, Libo Zhu

**Affiliations:** Department of Orthopaedics, Shanghai General Hospital, Shanghai, 201620 China

**Keywords:** Direct anterior approach, Hip, Arthroplasty, Component positioning

## Abstract

**Background:**

Controversy remains over whether different surgical approaches exert an impact on the component positioning in total hip arthroplasty. We conducted a retrospective study to reveal the long-term position of prostheses in the first group of patients in China who underwent direct anterior hip arthroplasty.

**Methods:**

Collected were data from 350 patients who underwent direct anterior hip arthroplasty between 2008 and 2013, including demographic information, imaging data, Harris hip scores, and surgical complications. Variables, measured radiographically or by CT, included hip offset, leg length discrepancy, component position, and stability within one week after surgery and at the last follow-up. The data were subjected to statistical analysis by using paired *t*-tests and Pearson chi-square tests.

**Results:**

Data were harvested by follow-up and self-reported questionnaires. The postoperative follow-up lasted for 13.1 years on average (minimum, 10 years; maximum, 15 years), and the overall survival rate of hip prostheses was 96.3%. The mean Harris score at the final follow-up was 91.8 points. After excluding patients with significant preoperative hip deformities, the incidence of postoperative limb inequality (> 5 mm) was 4.9% at the last follow-up, and the incidence of hip offset discrepancy (> 5 mm) was 14.6%. The overall proportion of the acetabular components located in the Lewinnek safe zone was 77.7%, whereas the proportion of femoral prostheses in the safe zone (< 3° inclination) was 94.0%. Based on the revised data and the last follow-up imaging, the total proportion of acetabular and femoral prostheses with a radiolucence of > 2 mm was 5.1%.

**Conclusion:**

Direct anterior approach hip arthroplasty could achieve excellent component positioning and long-term prosthesis survival in patients without severe hip deformities.

**Supplementary Information:**

The online version contains supplementary material available at 10.1186/s42836-024-00249-z.

## Introduction

Over the past decades, direct anterior hip arthroplasty has been recognized as a minimally invasive technique performed through the muscle interval and clinically achieved outstanding results [1–3]. Though many related reports have been published, long-term follow-up results of direct anterior hip arthroplasty are relatively limited compared to other techniques, such as surgeries via posterolateral or anterolateral approaches. In China, surgeons incrementally employed this minimally invasive hip replacement technique since 2008. However, it was not until approximately 2015 that more medical institutions began performing direct anterior approach hip arthroplasty. Given the excellent clinical results achieved in developed countries, the use of direct anterior hip arthroplasty has been rapidly on the rise in China. Nonetheless, according to statistical data from the Orthopedics Branch of the Chinese Medical Association, as of 2020, only roughly 8.3% of joint surgeons in China used this approach [4], which is far lower than the rates in developed countries, such as the United States [5].

Multiple factors contributed to this low application rate, including a steep learning curve [6, 7], doubts about the superiority of the new technologies (since traditional surgeries are good enough), and especially concerns about the accuracy of the prosthesis position. Some joint surgeons are concerned about whether the difficulty associated with the direct anterior approach can lead to poor placement of the prosthesis, leading to a series of surgical complications [8, 9]. However, controversy remains over whether direct anterior hip arthroplasty can attain a better hip prosthesis position than other techniques, and whether it can maintain a good prosthesis position on a long-term basis [10–13]. Our institute is one of the earliest in China to introduce the direct anterior approach hip arthroplasty and has been performing the procedure for over 15 years. In this study, we retrospectively looked at the component positioning and reviewed the long-term results of direct anterior hip arthroplasty based on imaging data from follow-ups spanning 10–15 years.

## Materials and methods

### Patients

This retrospective study was approved by the institutional ethics committee (No. 2023416). Informed consent was obtained from all subjects included in the study. A total of 495 cases of unilateral direct anterior hip arthroplasty performed at our hospital from January 2008 to December 2013 were retrospectively included, with 31 cases excluded due to loss of follow-up and 48 cases removed due to incomplete follow-up data. Sixty-six cases were also eliminated for being diagnosed as having type III and type IV hip dysplasia, infectious hip arthritis, ankylosing hip, obvious anatomical deformities, and leg length discrepancies before surgery. A total of 350 patients who underwent direct anterior hip arthroplasty were included in this study. They included 174 cases of aseptic necrosis of the femoral head, 85 cases of type I or II dysplasia and borderline dysplasia of the hip joint with osteoarthritis, 48 cases of femoral neck fractures, 23 cases of hip osteoarthritis, and 20 cases of rheumatoid arthritis. The longest follow-up period was 15 years, and the shortest lasted 10 years, with an average of 13.1 years. All patients were implanted with uncemented prostheses, with 117 patients using Stryker’s Accolade II femoral stem and Trident acetabular cup (Stryker, Mahwah, NJ, USA) and 233 receiving Zimmer’s M/L taper femoral stem and Trilogy or TM acetabular cup (Zimmer, Warsaw, IN, USA). Two senior doctors performed the direct anterior hip arthroplasty in all 350 patients. Additional patient data are presented in Table 1.

**Table 1:** Characteristics of patients

Category	Value
Gender	
Male	203
Female	147
Age (year)	65.9 ± 9.9
BMI	26.4 ± 2.8
Harris score	
Preoperative	57.8 ± 7.2
Last follow-up	91.8 ± 2.0
Operation time (minutes)	94.0 ± 14.7
Length of stay (days)	9.4 ± 1.6
Major complication	
dislocation	9 (7 revisions)
periprosthetic fractures	2 (1 revision)
infection	2 (2 revisions)
aseptic loosening	3 (3 revisions)

### Surgical technique

The patient was in a supine position and under general anesthesia. Taking the ASIS as a marker, a longitudinal incision of approximately 8 cm long was made along the longitudinal axis of the femur laterally toward the knee joint, starting about 2 cm outside and 2 cm distal to the ASIS. The fascia of the tensor fasciae latae was cut longitudinally and the muscle fibers of the tensor fasciae latae were separated from the medial muscle fascia by fingers and pulled to the outside. After careful separation and electrocoagulation of the lateral femoral circumflex vessels, the deep fascia tissue was opened to expose the fat layer in front of the hip joint. The joint capsule was cut open and two blunt retractors were used to encircle the femoral neck for osteotomy and then the femoral head was removed. The acetabulum was exposed, and the prosthesis was implanted under fluoroscopic control. After the proper release of the posterolateral capsule on the femoral side, we adducted, externally rotated, extended the affected limb, and inserted a retractor from behind the greater trochanter to lift the proximal femur. Upon preparation of the femur, a suitable prosthesis was implanted. After fluoroscopic examination and testing of limb length and joint stability, the fascia of the tensor fasciae latae and skin incision were sutured continuously.

### Design

**Fig. 1:**
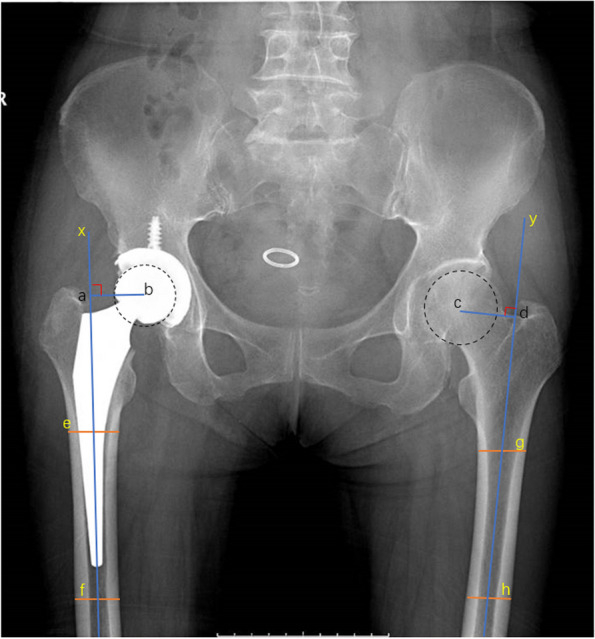
Schematic diagram of hip offset measurement on the operated and healthy sides. The line segments “ab” and “cd” represent the offsets on both sides, respectively

**Fig. 2:**
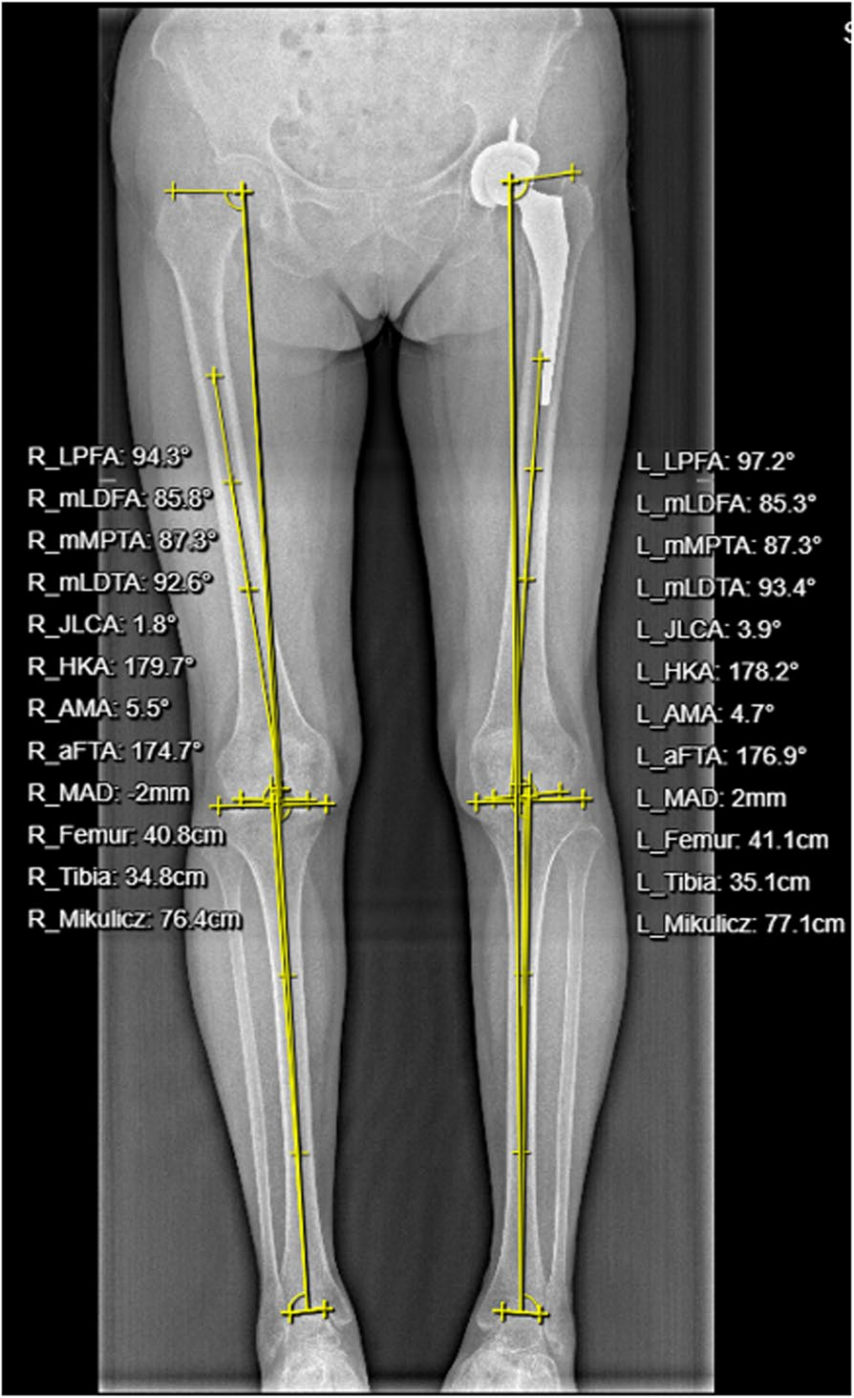
Schematic diagram for measuring the length of both lower limbs. The distance from the center of the femoral head to the highest point of the center of talus

**Fig. 3:**
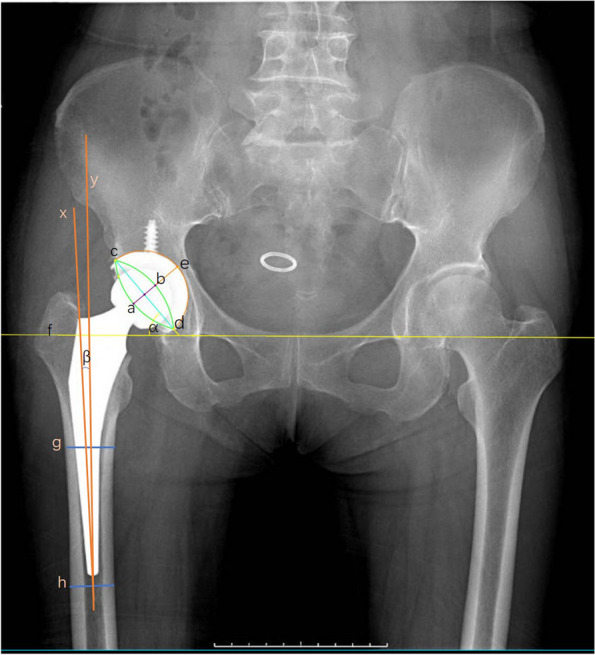
Widmer’s method (Anteversion = 48.05 × ab / ae – 0.3) measures the anteversion of the acetabulum on X-rays. “α” and “β” represent the abduction angle of the acetabulum and the eversion angle of the femoral stem, respectively. “X” is the longitudinal axis of the femoral stem, and “Y” is the longitudinal axis of the proximal femoral cavity

**Fig. 4:**
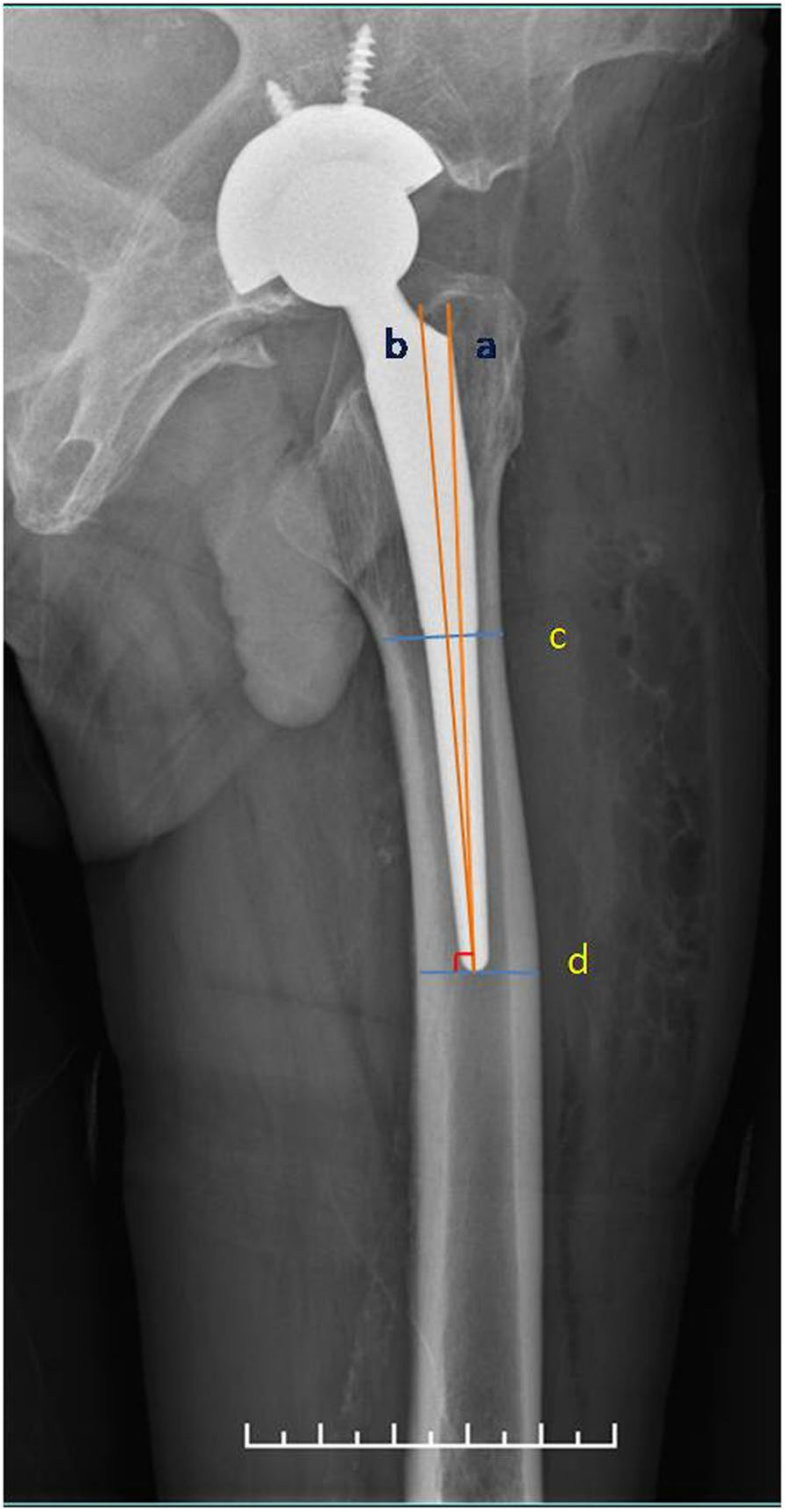
Line segments a and b represent the axes of the femoral prosthesis and the proximal femoral cavity, and the angle between them represents the alignment of the prosthesis on the sagittal plane

Based on postoperative X-ray films (Figs. 1, 2, 3 and 4) at one week and the last follow-up, acetabular component inclination, femoral stem alignment on the coronal and sagittal planes, bilateral hip joint offset, and length of both lower limbs were measured independently by two researchers. The radiolucence of all prostheses was also radiographically evaluated. Alignment of the femoral prosthesis on the coronal and sagittal planes was measured in terms of the angle subtended by the axis of the prosthesis and the axis of the proximal femoral cavity on X-ray. CT data of 143 patients were available, and their acetabular anteversion was directly measured. For the other 207 patients without CT data, the acetabular anteversion was measured by X-ray and converted by using Widmer’s method. While taking a plain film of the pelvis in the supine position, the radial bulb tube was tilted 10° from the ground perpendicular to the head, and both lower limbs were rotated 15° inward. Full-length films of both lower limbs were digitally used to synthesize images in three segments. The postoperative offset of the hip joint on the surgical side and the lengths of both lower limbs were compared, with the non-surgical side serving as a control. Lewinnek criteria define the acetabular component safe zone as an abduction angle of 40° ± 10° and an anteversion angle of 15° ± 10°. In this study, we defined the femoral component safe zone as an angle of ≤ 3° in both the coronal and sagittal planes. A difference of < 5 mm in the absolute length between the two lower limbs was taken as essentially equal in length. A deviation of < 5 mm between the operated and healthy sides was considered a return to the normal offset. During the follow-up period, the presence of a continuous 2 mm or more of radiolucence or subsidence around the prosthesis was seen as evidence of loosening.

### Statistical analysis

Mean and standard deviation were used to express continuous variables. ICC (Interclass Correlation Coefficient) was utilized to evaluate the consistency of measurement results of the same researcher and between different researchers. Changes in the acetabular anteversion and inclination angle between one week after surgery and the last follow-up were compared using a paired sample *t*-test for statistical analysis. The comparison between the proportion of the acetabular safe zone, offset, leg length discrepancy, and proportion of femoral component inversion was made by employing the Pearson Chi-square test. SPSS 26.0 was used for statistical analysis, and statistical significance was set at *P* < 0.05.

## Results

**Fig. 5:**
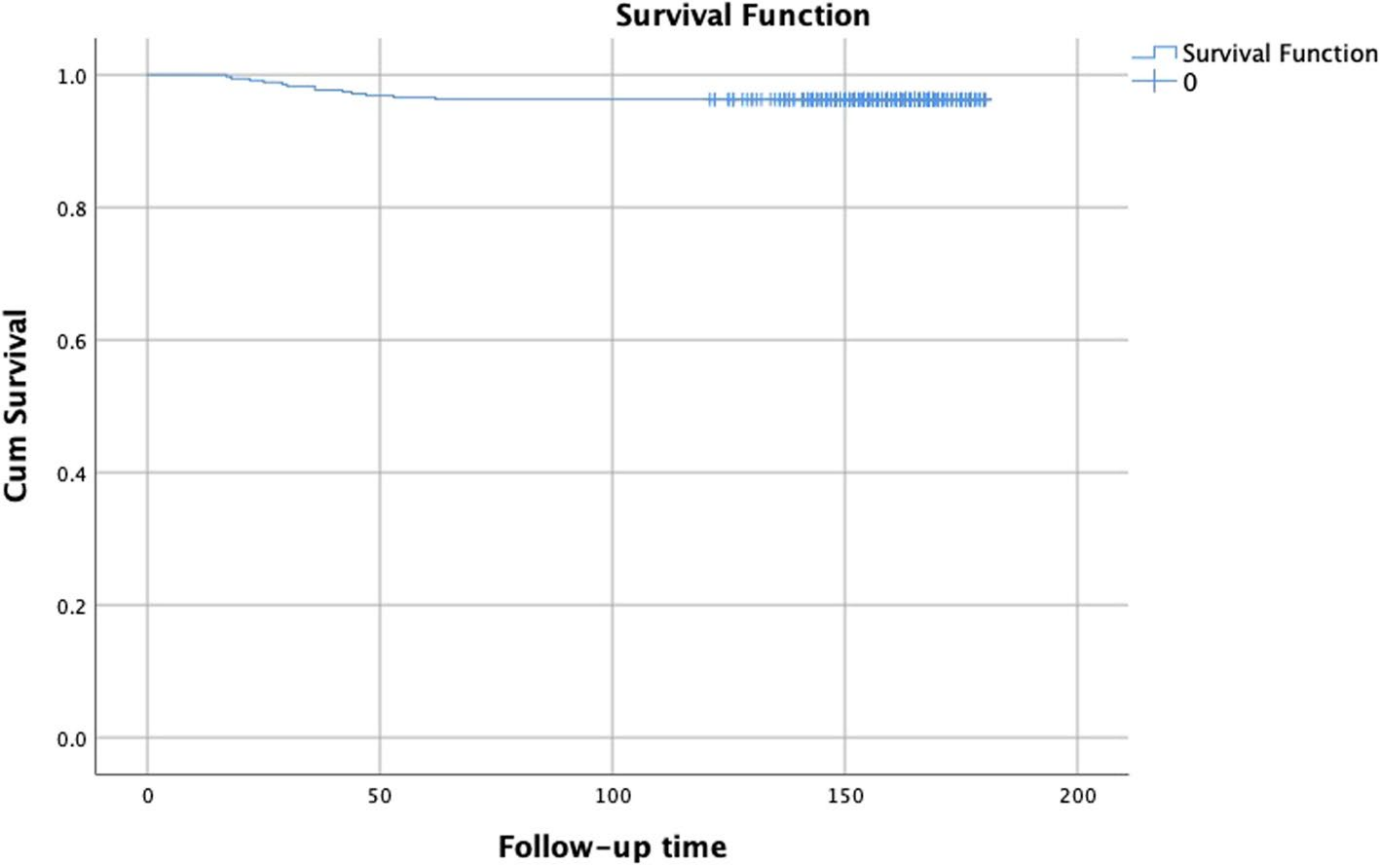
The overall survival rate of 350 cases of direct anterior approach hip arthroplasty in this group (SPSS 26.0, Kaplan–Meier analysis)

Sixteen patients in this group developed severe complications, of which 13 underwent revision surgery for dislocations (*n* = 7), aseptic loosening (*n* = 3), infections (*n* = 2), and periprosthetic fracture (*n* = 1). The overall survival rate of patients with hip prostheses was 96.3% (Fig. 5). Two cases of postoperative dislocation were treated with manual reduction and conservative treatment, whereas one case of periprosthetic fracture was treated conservatively, and healing was achieved. Data such as acetabular anteversion, inclination angle, hip offset, and femoral component orientation were obtained by measurements on plain pelvic films, femoral anteroposterior and lateral view films, full-length films of both lower limbs and hip CT scan images within one week after surgery and at the last follow-up (or revision), as shown in Tables 2 and 3.

**Table 2:** Analysis of imaging data of acetabular prosthesis

Category	One week	Intra-/inter-observer ICC	Last follow-up	Intra-/inter-observer ICC	*P* value
Anteversion (°)	17.3 ± 5.3	0.638/0.560	17.5 ± 5.3	0.644/0.573	0.324
Inclination (°)	43.9 ± 6.6	0.661/0.729	44.1 ± 6.5	0.711/0.750	0.303
Lewinnek safe zone	79.4%		77.7%		0.580
Offset discrepancy					0.584
≤ 5 mm	304		299		
> 5 mm	46 (13.1%)		51 (14.6%)		
Radiolucent (> 2 mm)	0		10		NA

**Table 3:** Analysis of imaging data of femoral prosthesis

Category	One week	Intra-/inter-observer ICC	Last follow-up	Intra-/inter-observer ICC	*P* value
Coronal plane		0.692/0.734		0.697/0.732	0.225
Valgus or Varus ≤ 3°	336		329		
Valgus or Varus > 3°	14		21		
Sagittal plane		0.719/0.693		0.706/0.684	0.320
Angulation < 3°	318		310		
Angulation > 3°	32		40		
Leg length discrepancy					0.732
≤ 5 mm	331		333		
> 5 mm	19		17		
Radiolucent (> 2 mm)	0		8		NA

The radiographic results of this group showed that the proportion of acetabular and femoral implants located within the safe zone was excellent, being 79.4% and nearly 90%, respectively, and this proportion remained highly consistent at follow-up after an average of 13 years. Moreover, over 80% of cases had returned to normal hip offset (difference of < 5 mm), and the proportion of bilateral lower limbs that were basically equal in length (difference of < 5 mm) was as high as 94%. Although 10 cases of acetabular prostheses and 8 femoral prostheses showed radiolucent lines (> 2 mm) during follow-up, only three of them eventually developed symptoms and required revision surgery.

## Discussion

Regardless of the surgical approach used, prosthesis position is very important for hip arthroplasty. It plays a crucial role in maintaining the stability of an artificial joint in the early stages, whereas in the long term, it is related to the survival of the component [14]. Simultaneously, restoring the normal rotation center and offset of the hip, as well as the length of both lower limbs, is crucial for restoring hip function and maintaining long-term limb mobility [15]. There exists still controversy regarding whether the direct anterior approach can achieve better postoperative prosthetic position and long-term prosthetic survival [16–18].

Although the “absolute safety” of Lewinnek’s safe zone has been questioned [19], a large amount of clinical practice has shown that it still serves as a standard for determining the position of acetabular implants after hip arthroplasty. In cases where there is no significant deformity of the joint before surgery, or there is no fusion in the spine [20], the Lewinnek safe zone remains the most commonly used evaluation system [21]. Previously, there has been significant controversy regarding whether the direct anterior approach can achieve a better acetabular position than other commonly used surgical approaches [22–24]. Through a multicenter prospective study, Gromov et al. found that the direct anterior approach had the highest proportion of acetabular prosthesis positions in the safe zone and the lowest variability in acetabular anteversion and inclination angle when compared to three other surgical approaches. Therefore, they believed that the direct anterior approach could accomplish a more stable and superior acetabular position [22]. However, Maeda and Goyal et al. found no difference in the component position between the direct anterior approach and other surgical approaches [18, 25]. Some authors have warned that a direct anterior approach may increase the anteversion angle of acetabular implants, posing the risk of anterior dislocation [10]. However, this scenario may be related to the experience of the surgeon or the learning curve of the technique, which can theoretically be resolved by experience accumulation or application of other navigation tools [26, 27]. Our follow-up data revealed that although approximately 20% of the acetabular prostheses were not within the safe zone, this result was still significantly lower than the level reported in a large population of patients undergoing posterior approach [28], with an overall postoperative dislocation rate of 2% and a long-term survival rate of 96.3%. This demonstrates that the direct anterior approach can achieve excellent prosthesis positioning and long-term survival. In addition, the restoration of hip offset is crucial for maintaining good abductor function and joint stability. In this group, 13%–14% had postoperative offset differences of > 5 mm from the normal side, which is similar to the results reported by other studies [29, 30]. Although it does not cause postoperative joint instability or dislocation, whether this difference in offset can cause postoperative gait and functional abnormalities remains controversial, and further research is warranted.

It is generally believed that the proportion of femur-related complications after direct anterior approach surgery may be higher than that for other surgical approaches [31]. Technically, exposure and operation of the femoral side through the direct anterior approach is relatively difficult and prone to intraoperative femoral perforation, prosthesis inversion, and femoral fracture. The probability of such complications was higher as compared to other approaches. However, this is clearly related to the early learning curve, as confirmed by Nairn’s report [6]. Studies have also reported that the direct anterior approach is more prone to early loosening of the prosthesis and sagittal malpostion [8, 32]. According to our follow-up results, although the incidence of malposition of the femoral component stood somewhere between 6%–12%, the proportion of periprosthetic fractures and radiolucent occurring 10 years after surgery was approximately 2.3%, which is significantly lower than that reported by other authors [33] and did not increase the revision rate. Through appropriate management, such as posterior lateral capsule release and piriformis tendon or external rotator tendon release, as well as the use of traction beds, the exposure of the proximal femur can be improved, and the risk of complications can be lowered. An over 10-year postoperative follow-up in our series showed that direct anterior hip arthroplasty could achieve good long-term stability and high survival rates of femoral prostheses. The proportion of postoperative leg-length discrepancies was also relatively low in this group. According to these data, the proportion of length discrepancy exceeding 5 mm was approximately 5%, which is significantly lower than that reported in the literature [34].

This study was subject to some limitations. Firstly, cases of severe anatomical lesions of the hip joint were excluded. Therefore, this study could not conclude that a direct anterior approach can also achieve a high long-term survival rate in patients with severe hip deformity. Another limitation was the deviation in image measurement and calculation. Though researchers in our group separately measured and analyzed the imaging data of these cases, some measurements were based on X-rays, whereas others were based on CT scans, which might result in inconsistencies in the measured angles. This may have led to differences between the results of this study and the findings reported by other studies.

## Conclusions

This study reported the imaging follow-up results of direct anterior hip arthroplasty in a Chinese population over a period time spanning 10–15 years. These results indicated that the direct anterior approach could achieve an excellent prosthesis position and long-term survival in patients without severe hip deformities. In the current context, where the usage rate in China is not very high, this approach has shown promise of being further promoted and more extensively employed.

### Supplementary Information

**Supplementary Material 1.**

## Data Availability

The datasets used and analyzed in the current study are available from the corresponding author on reasonable request. All data generated or analyzed during this study are included in this published article and its supplementary information files.
