# Supplementary figures and images for: Radiographic study of direct anterior approach hip arthroplasty: a 10–15 year follow-up of Chinese patients
Source: Arthroplasty. 2024 May 3;6:25. doi: 10.1186/s42836-024-00249-z (PMC11067169; doi:10.1186/s42836-024-00249-z)

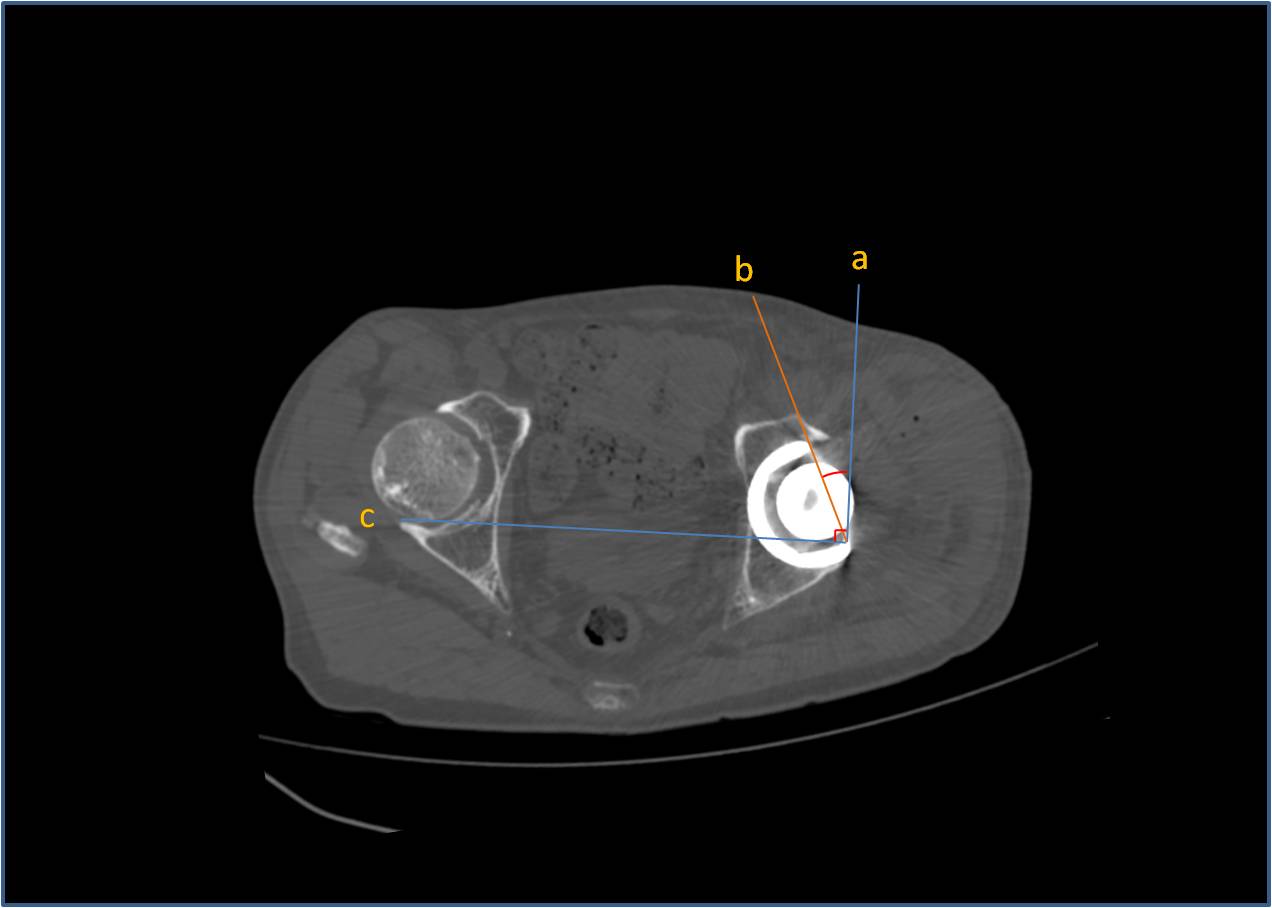

Supplement: Supplementary file 1 — Supplementary Material 1. [file 42836_2024_249_MOESM1_ESM.jpg]
